# Life Course Digital Twins–Intelligent Monitoring for Early and Continuous Intervention and Prevention (LifeTIME): Proposal for a Retrospective Cohort Study

**DOI:** 10.2196/35738

**Published:** 2022-05-26

**Authors:** Madison Milne-Ives, Lorna K Fraser, Asiya Khan, David Walker, Michelle Helena van Velthoven, Jon May, Ingrid Wolfe, Tracey Harding, Edward Meinert

**Affiliations:** 1 Centre for Health Technology University of Plymouth Plymouth United Kingdom; 2 Department of Health Sciences University of York York United Kingdom; 3 School of Engineering, Computing, and Mathematics University of Plymouth Plymouth United Kingdom; 4 Nuffield Department of Primary Care Medical Sciences Division University of Oxford Oxford United Kingdom; 5 School of Psychology University of Plymouth Plymouth United Kingdom; 6 Institute for Women’s and Children’s Health King’s College London London United Kingdom; 7 School of Nursing and Midwifery Faculty of Health University of Plymouth Plymouth United Kingdom; 8 Department of Primary Care and Public Health School of Public Health Imperial College London London United Kingdom; 9 Harvard T.H. Chan School of Public Health Harvard University Boston, MA United States

**Keywords:** artificial intelligence, machine learning, mulitmorbidity, mental health, health care, AI, outcome, NCDS, national child development study

## Abstract

**Background:**

Multimorbidity, which is associated with significant negative outcomes for individuals and health care systems, is increasing in the United Kingdom. However, there is a lack of knowledge about the risk factors (including health, behavior, and environment) for multimorbidity over time. An interdisciplinary approach is essential, as data science, artificial intelligence, and engineering concepts (digital twins) can identify key risk factors throughout the life course, potentially enabling personalized simulation of life-course risk for the development of multimorbidity. Predicting the risk of developing clusters of health conditions before they occur would add clinical value by enabling targeted early preventive interventions, advancing personalized care to improve outcomes, and reducing the burden on health care systems.

**Objective:**

This study aims to identify key risk factors that predict multimorbidity throughout the life course by developing an intelligent agent using digital twins so that early interventions can be delivered to improve health outcomes. The objectives of this study are to identify key predictors of lifetime risk of multimorbidity, create a series of simulated computational digital twins that predict risk levels for specific clusters of factors, and test the feasibility of the system.

**Methods:**

This study will use machine learning to develop digital twins by identifying key risk factors throughout the life course that predict the risk of later multimorbidity. The first stage of the development will be the training of a base predictive model. Data from the National Child Development Study, the North West London Integrated Care Record, the Clinical Practice Research Datalink, and Cerner’s Real World Data will be split into subsets for training and validation, which will be done following the k-fold cross-validation procedure and assessed with the Prediction Model Risk of Bias Assessment Tool (PROBAST). In addition, 2 data sets—the Early-Life Data Cross-linkage in Research study and the Children and Young People’s Health Partnership randomized controlled trial—will be used to develop a series of digital twin personas that simulate clusters of factors to predict different risk levels of developing multimorbidity.

**Results:**

The expected results are a validated model, a series of digital twin personas, and a proof-of-concept assessment.

**Conclusions:**

Digital twins could provide an individualized early warning system that predicts the risk of future health conditions and recommends the most effective intervention to minimize that risk. These insights could significantly improve an individual’s quality of life and healthy life expectancy and reduce population-level health burdens.

**International Registered Report Identifier (IRRID):**

PRR1-10.2196/35738

## Introduction

### Background

Multimorbidity, the co-occurrence of 2 or more long-term conditions, has been associated with numerous adverse outcomes and overwhelming financial burdens on health care systems [1]. Its prevalence in the United Kingdom is projected to almost double by 2035, and at least two-thirds of the gain in life expectancy for people aged >65 years will be spent with 4 or more chronic conditions [2]. Multimorbidity is most common in older people, but it is seen in patients of all ages [3]. It is more prevalent, and at younger ages, among people who live in socioeconomically deprived areas [4]. Health, behavioral, and environmental factors significantly influence individuals’ risk and many processes that lead to multimorbidity begin with chronic illness at much earlier ages. Multimorbidity can occur in clusters with predictable disease pathways [5]; however, the specialization of health care has created a challenge for identifying and treating co-occurring health conditions. An interdisciplinary approach combining clinical knowledge of disease pathways, clusters, and risk factors with artificial intelligence (AI) technology could improve understanding of the biosocial factors that are associated with developing multimorbidity. Understanding key risk factors for multimorbidity could enable doctors to monitor and treat patients more effectively, potentially mitigating or preventing multimorbidity.

### Rationale

A holistic, patient-centered system will be essential to address the challenge of preventing and managing multimorbidity and will require a combination of prediction, monitoring, and intervention. Digital twins—an engineering concept that uses real-world data and AI to provide a virtual representation of a physical counterpart—are starting to be explored in health care. The concept of the digital twin can be interpreted in relation to patients as a means of improving diagnostics and treatments by processing vast amounts of data to develop predictive health trajectories for individuals [6]. Our aim is to adapt this engineering solution to draw on continuously updated individual data on factors and health outcomes to simulate an individual’s future health status [7].

There is little established evidence on effective means of identifying risk factors or preventing and managing multimorbidity throughout the life course [8,9]. The vast majority of multimorbidity studies aim to identify disease clusters at a single point in time, providing little information about how multimorbidity develops over time within individuals [10]. This study will develop a system where innovative AI approaches are used to analyze complex longitudinal data and predict risk levels of multimorbidity. Using simulation, digital twins could identify key risk factors, consider weaknesses in source data through quantified underreporting, model potential adverse health outcomes, and recommend the most effective intervention when given adequate information. The use of digital twins is aimed at making significant enhancements within health care and advancing knowledge of disease [6]. When used in conjunction with clinician experience and knowledge, it will aid decision-making and highlight risk factors early on, so that steps can be taken to mitigate them and avoid later health consequences. The proposed digital twin solution has the potential to have a significant positive impact on individuals and health care systems.

### Aim, Hypothesis, and Objectives

The aim of this study is to identify key risk factors that predict multimorbidity throughout the life course by developing an intelligent agent using digital twins. The hypothesis is that this intelligent agent can use a wide range of biosocial variables (including physical and mental health and behavioral, socioeconomic, relational, and environmental conditions throughout an individual’s life) to predict risk of multimorbidity.

The three main objectives of the Life Course Digital Twins–Intelligent Monitoring for Early and Continuous Intervention and Prevention (LifeTIME) study are the following:

1. Identify key indicators that most accurately predict lifetime risk of developing multimorbidity.
2. Implement AI via a digital twin to simulate factors impacting people throughout their life to identify when they are at risk of later developing multimorbidity, enabling early, preventive interventions based on the critical indicators collected through patient-generated monitoring and medical records.
3. Evaluate the feasibility of the digital twin system by assessing the validity and performance of the predictive model.

## Methods

### Study Design

Using an implementation science theoretical framework and a retrospective cohort design, this will be a feasibility study aiming to address the following research question: Can historical data and data captured via a dynamic remote monitoring system be used to develop a digital twin that can predict individual risk of developing multimorbidity over a lifetime? An implementation science framework provides guidance regarding design and conduct to translate innovative ideas into practice [11]. It also gives ideas and suggestions to achieve various intended research pursuits.

**Figure 1:**
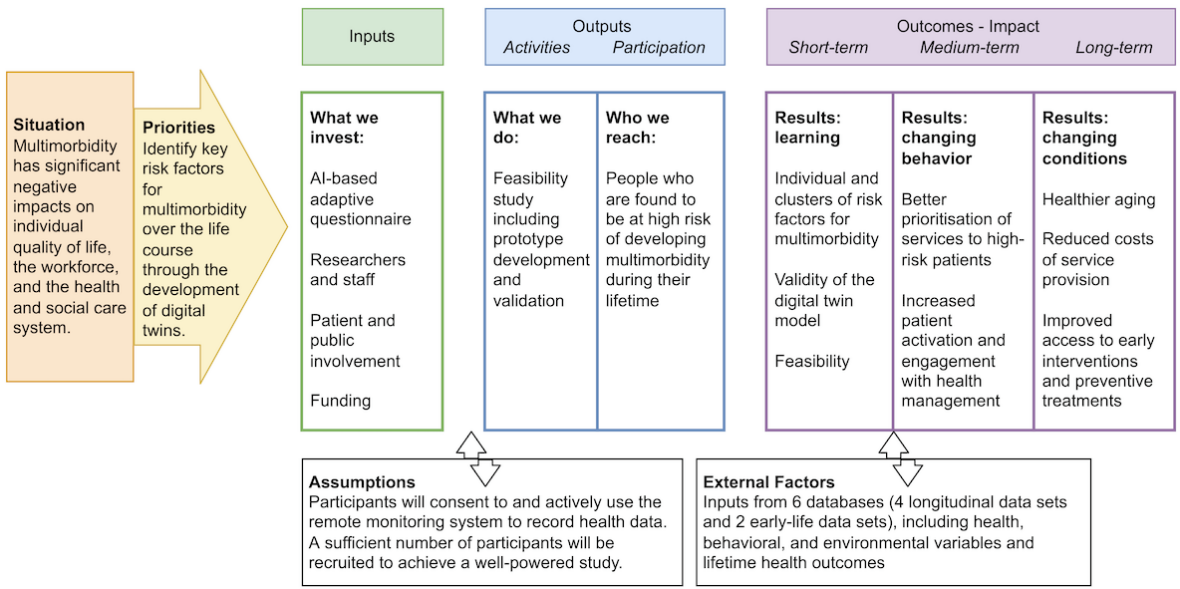
Life Course Digital Twins–Intelligent Monitoring for Early and Continuous Intervention and Prevention (LifeTIME) study logic diagram. AI: artificial intelligence.

For this study, 4 longitudinal databases will be used to determine how the presence or absence of particular factors (health, behavioral, and environmental) during childhood is associated with the development of health conditions and multimorbidity over the life course. The longitudinal data are needed to inform the model about childhood factors and health outcomes over the life course to identify potential early predictors of later multimorbidity. The study will consist of two phases: (1) prototype development, where the model will be trained to identify associations using subsets of the data sets; and (2) validation, where the model will be tested on the remaining subsets. Textbox 1 and Figure 1 provide an overview of the study design and framework.

**Population, Intervention, Comparator, and Outcomes (PICO) framework.**

**Population**

- Data for system development will be drawn from all age groups

**Intervention**

- An intelligent agent using digital twins that includes a dynamic remote monitoring system and a general predictive model

**Comparator**

- Real-world evidence via longitudinal data collected from databases will provide a means of validating the predictions made by the model

**Outcomes**

- Predicted risk of developing health conditions and multimorbidity over the lifetime

### Data Collection

The health care data that will be used to train and validate the model will be collected from several pre-established databases. To satisfy the data requirements of model training and validation, a few longitudinal data sets will be used and a variety of factors—including mental and physical health, and behavioral, environmental, and socioeconomic indicators—will be examined. The cohort used to train and validate the model will be built using data from previously established databases and split into random subsets. It will build upon previous work conducted on data linkage, the harmonization of multiple sources of patient-related e-records [12], and use of AI for health data analytics [13,14]. Using multiple databases will allow for a broader range of variables to be included in model development. In total, 4 longitudinal databases (the National Child Development Study [NCDS] [15], the Clinical Practice Research Datalink [CPRD] [16,17], the North West London Integrated Care Record [NWL ICR] [18], and Cerner’s Real-World Data from UK and Irish population [19]) were selected because they cover a range of mental and physical health outcomes, health behaviors, and other characteristics over decades in a diverse UK population (see Table 1).

**Table 1:** Characteristics of databases that will be used for model training and validation.

Database	Starting year	Number of patients	Location	Included data
National Child Development Study [15]	1958	17,415	England, Scotland, and Wales	Physical and educational development, economic circumstances, employment, family life, health behavior, well-being, social participation, and attitudes
Clinical Practice Research Datalink GOLD and Aurum [16,17]	GOLD: 1987; Aurum: 1995	GOLD: >11 million; Aurum: >19 million	GOLD: United Kingdom; Aurum: England (and Northern Ireland starting 2019)	Demographics, diagnoses, symptoms, signs, prescriptions, referrals, immunizations, behavioral and lifestyle factors, and tests
North West London Integrated Care Record Discover-NOW [18]	2015	>2.3 million	North West London	Data from all care settings (primary care, acute, mental health, community, and social care), for all disease areas
Cerner’s Real-World Data [19]	—^a^	~20 million	United Kingdom (30 trusts) and Ireland (7 hospitals)	Data recorded in electronic patient records

^a^Not available.

### Model and Digital Twin Development

Vast amounts of data will be used to develop the data sets, which will then generate a computational model to establish the relationship between biosocial factors and later health outcomes. Machine learning will be used to train a model using a variety of factors. The model will need to be trained on longitudinal data that includes both biosocial characteristics and later health conditions to establish links between factors and outcomes and account for historical differences in risk factor and multimorbidity prevalence.

**Figure 2:**
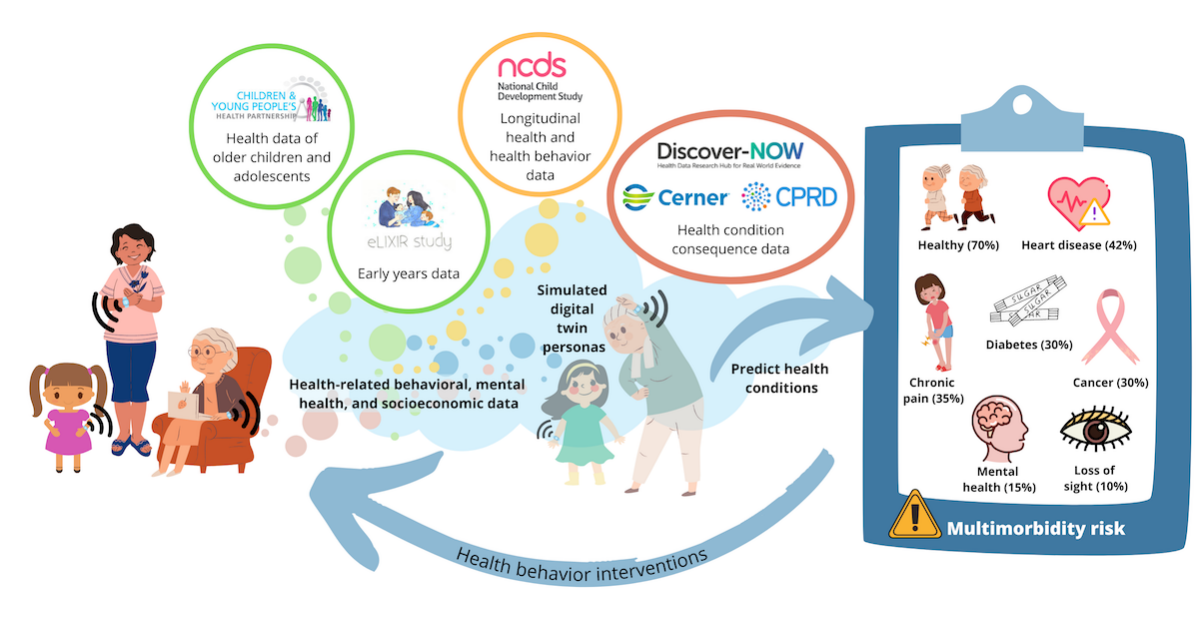
Life Course Digital Twins–Intelligent Monitoring for Early and Continuous Intervention and Prevention (LifeTIME) overview.

Once the predictive model has been developed based on the 4 longitudinal databases detailed in Table 1, two additional data sets will be incorporated into the development of the digital twins. Data from the Early-Life Data Cross-linkage in Research (eLIXIR) study [20] and the Children and Young People’s Health Partnership (CYPHP) randomized controlled trial [21] will be included in the development of the digital twins because they track early life variables and use of health care services. Using the preliminary model developed from the longitudinal data sets in combination with machine learning to identify clusters of factors that best predict the later risk of developing multimorbidity, a series of digital twin personas will be created to provide simulations of different types of people (see Figure 2).

Although the 2 additional data sets do not contain longitudinal health outcomes, incorporating them at this stage will enable the digital twin personas to be developed based on the clustering of a more comprehensive set of variables. For instance, if a particular variable was identified in the model as a predictor of multimorbidity, these additional data sets can provide more detail about variables that commonly co-occur with that predictor. This is beneficial because these data sets will provide more current data about early life factors than the longitudinal databases. The number of digital twin personas developed will depend on the clustering of the predictive factors. For this study, these personas will be developed to represent children, but in the long term, they will be developed to simulate different stages throughout life.

### Evaluation

The validity of the developed model and the digital twin prototypes will be assessed using the k-fold cross-validation procedure [22], with k being determined when the total cohort size is known. The machine learning algorithms will be tested on the validation data set to determine which model has the best fit. Model validity will be examined using a variety of indicators, including receiver operating characteristic and precision-recall curves (see Table 2). The Prediction Model Risk of Bias Assessment Tool (PROBAST) will be used to assess the model’s risk of bias [23].

**Table 2:** Primary and secondary objectives and outcomes.

Objective	Primary outcome	Secondary outcome
Identify key indicators that most accurately predict lifetime risk of multimorbidity	Risk factors for multimorbidity	N/A^a^
Assess the validity of a model that identifies variables and predicts lifetime risk of developing multimorbidity	Validity	Risk of bias

^a^N/A: not applicable.

### Ethics

Ethical approval for this study will be sought from the University of Plymouth’s Faculty of Health Research Ethics and Integrity Committee. All the data being used in this proof-of-concept study will be procured from pre-existing databases. Therefore, all data access will be dependent on the approval of the study protocol by the data controllers for those databases; for instance, the CPRD database requires that study protocols be approved by an Independent Scientific Advisory Committee before data are shared. All storage and use of the data provided will comply with database-specific and General Data Protection Regulation (GDPR) requirements. It will be confirmed before access that the data have been sufficiently anonymized to comply with GDPR requirements and that the databases’ patient consent processes extend to cover the sharing of anonymized data for other research purposes (ie, that their personal health data is being used in a way that could be reasonably expected by the participants). Processes for the removal of patient data—at their request to the specific database—will be established before data use with each of the database managers. Potential ethical issues relating to the development and implementation of digital twins in health care will be identified from the literature and discussions with a patient and public steering group.

## Results

The study is expected to produce a validated model of the factors associated with later development of mental health conditions and multimorbidity and a series of digital personas based on that model. The results collected will inform assessments of the feasibility of pursuing further development and evaluation of the digital twin system; if unfeasible in its current state, the findings will inform understanding of problems and iterative development. This study is a starting point; the results will provide data to inform a base model that will be used in later studies, where the model will be trained and developed using remote monitoring of and data collection from real patients in combination with population health data to more closely personalize the digital twins.

## Discussion

### Overview

By associating a patient with a digital twin persona and then personalizing it with their evolving data, health care providers and researchers could receive a personalized risk score representing the probability that the patient will develop further health complications. With the capability to start at a young age, a digital twin system has the potential to provide significant clinical value by identifying risk factors early, so that preventive interventions can be made to reduce an individual’s risk of developing long-term health conditions. This would save time and resources for health care providers, hospitals, and health care systems and reduce health problems and related financial costs for patients.

### Future Directions

If the study results are positive, future research could develop and incorporate a remote monitoring system that can collect and collate individual data with the population data in a pilot trial. This would enable the digital twins to be further developed and iteratively improved based on feedback from the participants. As the system will use sensitive personal data and have ethical implications, safety and efficacy data will be an essential next step on the path to achieving wide-scale patient benefit.

Based on a user’s personal data, health care providers and researchers would be able to associate an individual patient with the digital twin persona that is the best fit for them. This will then provide a risk score representing the probability that the patient will develop further complications or health conditions. In the future, the digital twin will also be trained with data on interventions so that it can provide recommendations to health care providers about what interventions are most likely to be effective for that individual. In the long term, data collected from this remote monitoring system will be combined with childhood digital twin personas to increase the variables for prediction and to personalize the best-fitting persona based on individual health data.

### Limitations

Potential limitations of the study have been identified, with plans for their mitigation, to maximize the potential usefulness of the study.

1. Data linkage: The data sets used in the study are from a variety of databases that were not designed to be integrated and were not collected from the same populations. This will be mitigated by the experience of the research team and the use of machine learning techniques to aggregate a large amount of data.
2. Data quality: Understanding the errors in data is key to making predictions. This can be more difficult to quantify for survey data (particularly subjective questions) than for technical medical data. This will be mitigated by collecting data from multiple sources with large sample sizes and by prioritizing the inclusion of validated questionnaires in future remote monitoring systems.
3. Lack of individualization: Developing a model based on pre-existing data sets that do not all follow specific individuals means that there is a risk that the “digital twins” will only be a predictive model. This will be mitigated by the future development of a novel and linked system of collecting remote monitoring and sensing data that can be incorporated into digital twin personas.

### Conclusion

Incorporating a remote monitoring system would enable tracking of ongoing lifestyle data, which could be combined with the individual’s personal health data and population health and lifestyle data to potentially increase the potential impact. Personalized patient simulations could be used to optimize prevention or treatment selection, saving patients unnecessary side effects, reducing the number of treatments attempted, and reducing the need for repeat hospital visits. Prevention and early intervention to manage multimorbidity will support healthy aging and reduce its negative impacts on quality of life and the workforce.

## Abbreviations

AI: artificial intelligence
CPRD: Clinical Practice Research Datalink
CYPHP: Children and Young People’s Health Partnership
eLIXIR: Early-Life Data Cross-linkage in Research
GDPR: General Data Protection Regulation
LifeTIME: Life Course Digital Twins—Intelligent Monitoring for Early and Continuous Intervention and Prevention
NCDS: National Child Development Study
NWL ICR: North West London Integrated Care Record
PROBAST: Prediction Model Risk of Bias Assessment Tool
